# Tomosynthesis-Detected Architectural Distortions: Correlations between Imaging Characteristics and Histopathologic Outcomes

**DOI:** 10.3390/jimaging9050103

**Published:** 2023-05-19

**Authors:** Giovanna Romanucci, Francesca Fornasa, Andrea Caneva, Claudia Rossati, Marta Mandarà, Oscar Tommasini, Rossella Rella

**Affiliations:** 1 UOSD Breast Unit ULSS9, Ospedale di Marzana, Piazzale Lambranzi, 1, 37142 Verona, Italy; 2 Department of Radiology, G. Fracastoro Hospital, 37047 Verona, Italy; 3 Division of Pathology, G. Fracastoro Hospital, 37047 Verona, Italy; andrea.caneva@aulss9.veneto.it; 4 Department of Oncology, ULSS9 Scaligera, G. Fracastoro Hospital, 37047 Verona, Italy; 5 UOC Diagnostica per Immagini, Ospedale G.B. Grassi, Via Gian Carlo Passeroni, 28, 00122 Rome, Italy

**Keywords:** architectural distortion, tomosynthesis, synthetic2D, B3 lesions, DBT-vacuum assisted biopsy, breast cancer, Breast Cancer Screening Program, breast imaging

## Abstract

Objective: to determine the positive predictive value (PPV) of tomosynthesis (DBT)-detected architectural distortions (ADs) and evaluate correlations between AD’s imaging characteristics and histopathologic outcomes. Methods: biopsies performed between 2019 and 2021 on ADs were included. Images were interpreted by dedicated breast imaging radiologists. Pathologic results after DBT-vacuum assisted biopsy (DBT-VAB) and core needle biopsy were compared with AD detected by DBT, synthetic2D (synt2D) and ultrasound (US). Results: US was performed to assess a correlation for ADs in all 123 cases and a US correlation was identified in 12/123 (9.7%) cases, which underwent US-guided core needle biopsy (CNB). The remaining 111/123 (90.2%) ADs were biopsied under DBT guidance. Among the 123 ADs included, 33/123 (26.8%) yielded malignant results. The overall PPV for malignancy was 30.1% (37/123). The imaging-specific PPV for malignancy was 19.2% (5/26) for DBT-only ADs, 28.2% (24/85) for ADs visible on DBT and synth2D mammography and 66.7% (8/12) for ADs with a US correlation with a statistically significant difference among the three groups (*p* = 0.01). Conclusions: DBT-only ADs demonstrated a lower PPV of malignancy when compared with syntD mammography, and DBT detected ADs but not low enough to avoid biopsy. As the presence of a US correlate was found to be related with malignancy, it should increase the radiologist’s level of suspicion, even when CNB returned a B3 result.

## 1. Introduction

Architectural distortion (AD) is defined as a distortive area without associated mass and is seen in mammography as thin “straight lines or spiculations which radiate from a point, and focal retraction, distortion or straightening at the anterior or posterior edge of the parenchyma” [1]. It is the third most common mammographic sign of non-palpable breast cancer, accounting for nearly 6% of abnormalities detected during screening mammography [1].

AD correlates with some pathological entities, both benign and malignant, such as postprocedural changes, radial scar, complex sclerosing lesion, fat necrosis or breast carcinoma [2,3].

Concerning breast carcinoma, although the prevalence of AD on mammography is lower compared to that of calcification or visible mass [1], it is shown to have a positive predictive value (PPV) for malignancy up to 75.5% [4]. Furthermore, it accounts for 12% to 45% of missed cancer at conventional mammography [5], representing a subtle finding on traditional 2D digital screening mammography and a diagnostic challenge for breast radiologists. The advent of 3D digital breast tomosynthesis (DBT), and its growing use in diagnostic and screening settings, has improved the detection of AD [6]. In particular, DBT reduces the superimposition of overlapping structures allowing AD’s detection. DBT has also been found to improve the visualization of AD even in low density breasts [7]. For these reasons, it is detected more frequently on tomosynthesis than on 2D digital mammography and can also be missed on conventional 2D imaging [3].

However, this increased detection rate has also translated to a decreased PPV: the calculated PPV for AD only seen in DBT is 34.6% [8]. As a consequence (as AD could refer both to benign, high-risk or malignant lesions), it is important to study the radiological—histological correlation between AD identified on DBT and the risk of malignancy to determine the correct diagnostic workup and management of these lesions.

The purpose of this study is to determine the positive predictive value (PPV) of DBT-detected ADs and evaluate the correlation between imaging characteristics and histopathologic outcomes.

## 2. Materials and Methods

### 2.1. Patient Selection

This prospective study was approved by our institutional ethics committee (clinical trial identification: 2019/47440) of Verona and Rovigo Province. Written informed consent was obtained for all women included in the study.

**Figure 1:**
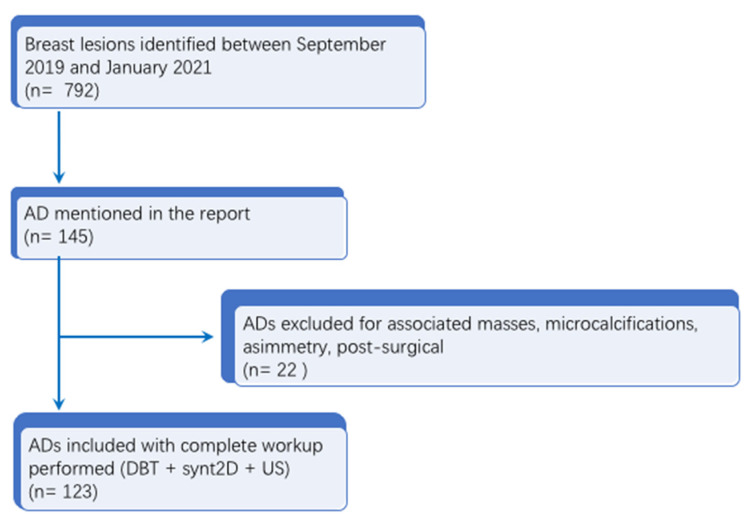
Flow chart of the study.

In this subset, the data from all patients with breast lesions who had undergone an invasive diagnostic procedure (CNB or DBT-VAB) at our institution from September 2019 to January 2021 were analyzed, for a total of 792 lesions. Of these 792 lesions, 145 patients with a report of architectural distortion (AD) were initially identified. Cases of AD with an associated mass, calcifications, asymmetry or post-surgical AD were excluded (*n* = 22). All the ADs included had synt2D, DBT and US images available. Malignant lesions or lesions of unknown biological potential (B3 lesions) were included only when surgical excision was performed at our hospital with histopathological examination of the entire lesion or when a radiologic follow-up (FUP) ≥24 months was available. Finally, 123 patients with 123 ADs were included in the analysis with a mean age at diagnosis of 59 ± 7.5 (SD) years (range: 50–74 years). Study flowchart is available in Figure 1.

### 2.2. Imaging Technique and Interpretation

Mammographic imaging was performed using a mammography unit (Selenia Dimensions, Hologic, Bedford, MA, USA). In our institution, all the women who participated in the Breast Cancer Screening Program underwent a DBT exam, in the context of a prospective study regarding DBT utilisation in the screening setting approved by an ethical committee. Screening examination include two views (craniocaudal and mediolateral oblique) DBT plus synthetic 2D mammography of each breast. Synthetic 2D mammography was reconstructed by the Hologic C-view algorithm. All images were interpreted in blinded double reading by two independent radiologists. Double reading were performed using dedicated workstations and were distributed across six breast radiologist pairs, each with at least 10 years of experience with breast imaging and 5 years of experience with DBT (G.R., P.B, L.C., A.V., L.Z., M.V.B.). A screening was considered positive and a woman recalled for second-level diagnostic work-up if at least one reader recorded a positive result. Screening examinations were classified to Breast Imaging Reporting and Data Sistem (BI-RADS) classification (2). BI-RADS 0 refers to an incomplete evaluation with further imaging required including additional views; BI-RADS 1 refers to a negative examination, with no findings; and BI-RADS 2 is consistent with benign findings, such as simple cysts, benign calcifications, fat-containing lesions, fibroadenomas, and intramammary lymph nodes. BI-RADS 3 is probably benign; and BI-RADS 4 is a suspicious abnormality, which can represent the chance of being malignant and is subdivided into three subcategories: a, b, and c. The subcategory of (a) has a low probability of malignancy with a 2% to 10% chance of malignancy. The subcategory of (b) has an intermediate change of malignancy ranging from 10% to 50%. The subcategory of (c) has a high probability of malignancy ranging from 50% to 95%. BI-RADS 5 is highly suggestive of malignancy. BI-RADS 6 is used for pathology proven malignancy.

All diagnostic work-up images for recalled women were interpreted by dedicated breast radiologists with more than 10 years experience in breast imaging and more than 5 years experience in DBT.

Patients recalled from screening for further evaluation (second-level assessment) could potentially undergo an additional mammographic/DBT imaging (magnification views or spot compressions in DBT), ultrasound (US) examinations, core biopsy under ultrasound guidance (US-guided CNB) or DBT-vacuum assisted biopsy (DBT-VAB), if indicated.

US examinations were performed using a GE LOGIQ E9 echograph (GE Healthcare, Chalfont St Giles, UK) or a Philips EPIQ 9 echograph (Andover, MA, USA) equipped with a 6.0- to 15-MHz and 5.0- to 18-MHz linear transducer, respectively, using a freehand positioning technique. US esaminations were performed by six breast radiologists with at least 5 years of experience. Images were interpreted in real-time during the second-level diagnostic work-up and biopsy was performed of all mass and non-mass findings that were visible at US.

Lesions classified as BI-RADS 4a, 4b, 4c (suspicious abnormality) or 5 (highly suggestive of malignacy) underwent a biopsy.

### 2.3. Biopsy Tecnique

DBT-VAB was performed using a platform that allows acquisition of three-dimensional tomosynthesis (Selenia Dimensions 3D; Hologic) and a C-View software which produces 2D images. For breast biopsy, a dedicated guidance system (Affirm; Hologic) was attached. DBT-VAB were performed with a 9-gauge vacuum biopsy device (Eviva; Hologic, Bedford, MA, USA) with an aperture of 20 mm or 12 mm. The patient was positioned in the upright position in a dedicated armchair. The biopsy approach was chosen on the basis of breast thickness and lesion location. DBT was performed to identify the target lesion and the DBT section with the best target visualization was chosen. When the operator indicated the position of the target, coordinates were automatically determined by the biopsy software system, including *z*-axis location. The system shows a graphic representation of the real situation: breast thickness and position of the target lesion. After skin disinfection, local anesthetic was induced with 10 mL of lidocaine (Lidosen, Galenica Senese Srl, Monteroni D’arbia, Siena, Italy). DBT was repeated to reidentify the target lesion to avoid errors due to the lesion movement subsequent to aesthetic injection and biopsy coordinates were recalculated. After inserting the biopsy needle, the pre-fire control was performed with two stereotactic images (+15° and −15°). In the study group, for architectural distortion, 12–24 biopsy specimens (mean weight 4 g) were obtained. Finally, post-fire control was performed with two more stereotactic images (+15° and −15°). After the biopsy, saline lavage was performed to reduce the rate of hematoma occurrence and to avoid the movement of the biopsy marker. A clip (Securmark for Eviva; Hologic) was placed in all patients and DBT was performed to check the clip position. At the end of the procedure, a compression bandage was placed. After seven days, two mammograms projections (cranio-caudal and latero-medial or mediolateral views) were obtained to check the clip position, document the target lesion removal and to quantify the residual lesion, if present.

US-guided core-needle biopsy, with a 14G needle, was performed using a freehand positioning technique. After skin disinfection, local anesthetic was induced with 5 mL of lidocaine (Lidosen, Galenica Senese Srl, Italy). Three to four specimens were obtained. At the end of the procedure, a compression bandage was placed. In this case, the clip was not positioned due to the small number of specimens; therefore, the lesion is still echo-visible for possible centering in the event of subsequent surgical removal.

### 2.4. Histopathological Evaluation

All the specimens were immediately placed in fixative solution (formalyn 10%), processed in the laboratory of Pathology and paraffin embedded; four 2-micron sections were obtained and stained with haematoxylin and eosin analyzed by a dedicated breast pathologist with 23 years of experience. Results were classified from B1 to B5, according to the European guidelines [9] which classify histopathological results as follows: B1, normal tissue/non-diagnostic, regardless of whether breast parenchymal structures are present; B2, benign abnormalities such as fat necrosis, ductal ectasia, intra-mammary limph nodes, sclerosing adenosis, fibroadneomas, fibrocystic changes; B3, category is a heterogeneous group of lesions of unknown biological potential and includes: (a) atypical intraductal epithelial proliferation (ADH); (b) flat epithelial atypia (FEA); (c) lobular intraepithelial neoplasia (LIN) including both atypical lobular hyperplasia (ALH) and lobular carcinoma in situ classic type with low or intermediate nuclear grade (LCIS); (d) radial scar/complex sclerosing lesion (RS/CSL); (e) papillary lesion (PL) and (f) “other entities” including fibroepithelial lesion with cellular stroma and “mucocele-like” lesion; B4, suspicious for malignancy but insufficient for a definitive diagnosis; and B5, malignant, which includes four subcategories: B5a, in situ carcinomas; B5b, invasive carcinomas; B5c, non assessable invasive status; B5d, other malignancy.

In malignant, high-risk, or discordant cases, subsequent localization and surgical excision were performed.

### 2.5. Data Collection and Statistical Analysis

Microhistological results were available for all needle biopsies, and surgical histological examinations were reviewed for lesions which underwent surgical excision.

Medical reports and histopathological analysis were reviewed to collect data. Radiology reports were reviewed to identify AD visualisation on DBT images and on synth2D mammography. Data were entered into a computerised spreadsheet (Excel; Microsoft, Redmond, CA, USA).

The positive predictive value (PPV) for malignancy (based on biopsy or surgical excision histopathological results or imaging FUP) was calculated as follows: PPV (%) = number of malignant lesions/total of lesions × 100.

The Chi-square test was used to analyse the association between imaging visibility of AD and histopathologic outcomes. A *p*-value < 0.05 indicated a statistically significant result.

## 3. Results

**Figure 2:**
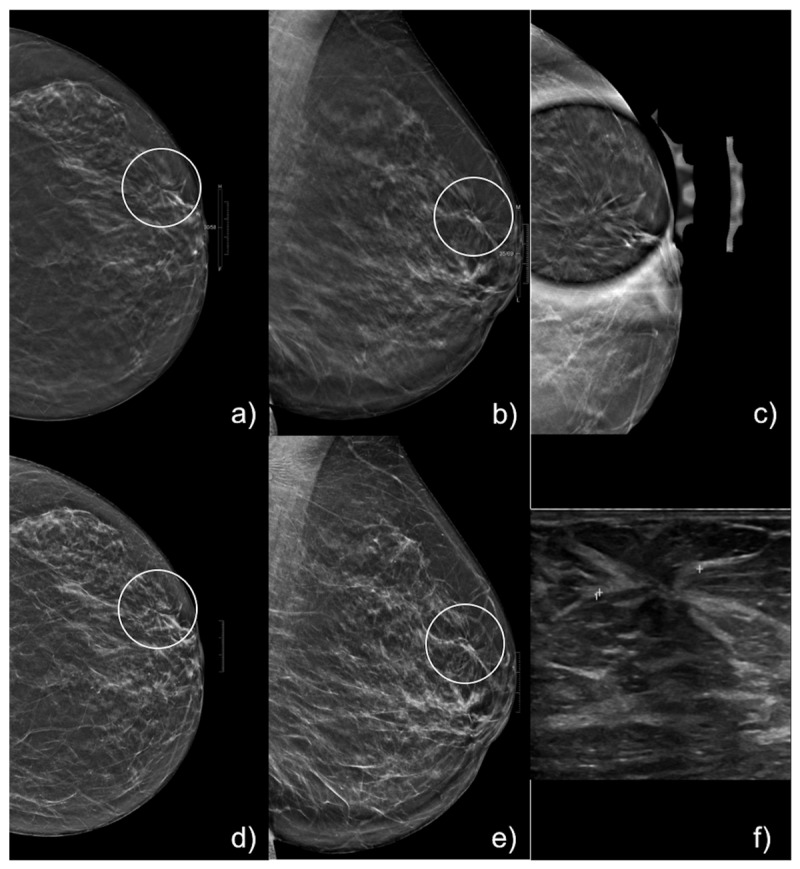
69 years old woman who presented for screening mammography with an AD of 25 mm, in left upper-external quadrant (circle). AD was fully appreciable on DBT images (**a**,**b**), on DBT spot compression (**c**) and on synt2D images (**d**,**e**). It showed US correlate (**f**), therefore US-CNB was performed. At histological analysis, a tubular carcinoma was found.

A total of 123 ADs underwent biopsy. Diagnostic US was performed to assess a correlate for ADs in all 123 cases and a US correlate was identified in 12/123 (9.7%) cases (Figure 2), which underwent US-guided CNB. The remaining 111/123 (90.2%) ADs were biopsied under DBT guidance. Histopathological correlation of only DBT and DBT + synt2D detected ADs are summarised in Table 1 while histopathological correlation of DBT + US detected ADs and for DBT + synt2D + US detected ADs are summarised in Table 2.

Among the 123 ADs included, 33/123 (26.8%) yielded malignant results, 68/123 (55.2%) B3 lesions and 22/123 (17.8%) benign results.

Among the 68 B3 lesions, 14/68 (20.5%) underwent surgical excision and 4/14 (28.5%) were upgraded to malignancy; the remaining 54/68 (79.4%) B3 lesions underwent radiological follow-up, all mammographically stable. The overall PPV for malignancy was 30.1% (37/123).

Of the 26/123 (21.1%) cases of DBT-only ADs (not seen on synth2D mammography)—all that were biopsied under DBT guidance—we found 3/26 B2 (11.5%), 18/23 B3 (69.2%) (all unchanged at radiological FUP and remained mammographically stable for an average of 3 years, range 2–4 years) and 5/23 B5 (19.2%) lesions. A total of 85/123 ADs were visible both on DBT and synt2D mammography and underwent DBT-guided VAB, yielding the following results: 16/85 B2 (18.7%), 47/85 B3 (57.5%) (two upgraded to malignancy after surgical excision) and 22/85 B5 (23.7%). Results from the surgical or excisional biopsy and the 2 year follow up for B3 to B5 upgrading of lesions are summarised in Table 3.

Of the 12/123 AD cases with a US correlate, which underwent US-guided CNB, 6/12 (50.0%) demonstrated malignant pathology: three B3 (33.3%) lesions (two upgraded to malignancy after surgical excision) and three B2 (66.6%). The imaging-specific PPV for malignancy was 19.2% (5/26) for DBT-only ADs, 28.2% (24/85) for ADs visible on DBT and synth2D mammography, and 66.7% (8/12) for ADs with a US correlate that meant a statistically significant difference among the three groups (*p* = 0.01).

## 4. Discussion

During recent years, the use of DBT has increased as a breast imaging method in diagnostic and screening contexts. The increase in the use of DBT in breast screening and in clinical practice has increased the sensitivity and specificity of the breast investigation with an increase for the detection of small cancers, a reduction in the rate of recalls in organized screening programs, and an increase in diagnostic accuracy. DBT use in diagnostic and screening contexts also increased the number of detected ADs especially for the minimization of tissue superimposition [8,10,11].

While AD can be due to malignancy, it is also a common presentation of radial scar and complex sclerosing lesions, both of which are lesions of uncertain malignant potential. Given the higher detection of ADs with DBT, more radial scars and complex sclerosing lesions are diagnosed, raising questions as to whether and when to biopsy an AD seen on DBT [8]. In our single-institution study, we found an overall PPV for malignancy of 30.1%, in line with previous published results [12].

In our series, 26/123 (21.1%) lesions were visible only on DBT images; this result underlines the fact that the advent and implementation of DBT needs to be associated with the availability of a DBT-guided biopsy system to correctly manage these lesions.

We also evaluated the correlation between imaging visibility of ADs and histopathologic outcome, and we found a statistically significant association between imaging method visibility and PPV for malignancy.

The PPV of malignancy resulted lower when AD was visible only on DBT images (19.2%), in line with results of previous studies (with PPVs ranging from 10.2% to 68.4% [10,13] but not low enough to avoid biopsy [12].

When ADs are visible both on DBT and synt2D (but without a US correlate), the PPV for malignancy increased to 28.2%. Thus, the detection of AD on synthetic mammography should raise the level of suspicion, as previously reported by Feliciano et al. [14].

Regarding a US correlate for ADs, we found a US correlate in only 12/123 (9.7%) ADs, a percentage lower than previous published results [15]. In our opinion this may be related to the exceptional level of DBT reading experience of our breast radiologists with more than 8000 DBT read every year since 2014. DBT use in the screening setting was approved by the ethical committee in our institution [13]. This leads to a more frequent detection of ADs, often without US correlate.

We found a significant association between the identification of an US correlate with AD and the presence of malignancy with a PPV of 66.7%. This result shows that a US correlate is a predictor of malignancy and is aligned with the results of previous published studies and metanalysis [7,16].

As reported in the previous studies, increased visibility of ADs on DBT (if compared with 2D conventional mammography) also leads also to an increased diagnosis of B3 lesions presenting as ADs [15]. In our study, lesions of uncertain malignant potential (B3 lesions) were higher in the DBT-VAB group than the CNB group (58.5% versus 25.0%, respectively). A correct management of patients with a B3 lesion diagnosis is mandatory. However, lesions of uncertain malignant potential of the breast show a underestimation in the literature from 9.9 to 35% after surgical excision of the lesion. In our series, analysing only B3 results, none of the DBT-only ADs yielding a B3 result were upgraded to malignancy (after surgical excision or imaging FUP) while 2/47 (4.2%) of B3 ADs visible both on DBT and synt2D and 2/3 (66.7%) of B3 ADs with a US correlate were upgraded to malignancy after surgical excision. These results may be related to the different biopsy techniques (VAB versus CNB) with different amounts of tissue for the pathologist to examine. These results suggest that when US-guided CNB is performed on AD, it returns B3 results; therefore, the lesion should be excised or biopsed via VAB with more cores to increase sampling accuracy and to minimize underestimation.

In our experience, DBT-VAB allowed using DBT in breast cancer screening in terms of higher breast cancer detection rate, limiting the potential drawbacks. Indeed, in our series, patients with architectural distortions and a B5 microhistological result have been treated with conservative surgery, permitted by the early detection of breast cancer (with small lesion sizes), with lower costs for cancer treatment [17]. On the other hand, the pathologist’s time required increases. With this new approach, the average number of blocks submitted are six with four sections on each block and a trimming at thickness of 30 μm, with a total of twenty-four sections stained with haematoxylin and eosin. A careful examination of the tissue on more sections allows for a more accurate evaluation of the lesion with the identification of possible small foci of atypical epithelial proliferation or lobular intraepithelial neoplasia. Moreover, the morphological detail on the sections is definitely better in the tissue obtained via the VAB technique with a 9G needle, than that obtained with the cutting needle used in the ultrasound-guided core needle biopsy with less crush artefacts in the first one. Therefore, the longer the pathologist’s time is required, the better quality of the samples in the VAB technique [18]. Additionally, when compared with surgical biopsy, the quality of the specimens obtained with VAB technique is better. Indeed, due to the non-malignant nature of these lesions, the surgical biopsy should excise a very small amount of breast tissue for cosmetic reasons. So, when the incision margins are not adequately away from the lesion, surgical biopsy could provide a sample with fulguration artefacts that compromise the samples interpretation and the histological diagnosis. The objections about the fragmentation of the lesion in the specimens obtained with the VAB technique are founded on nothing because of the larger dimensions of the cores obtained which enable the pathologist to make an accurate morphologic evaluation of the lesions, better than what is possible on the tissue provided by surgical biopsy [19]. For all these reasons, DBT-VAB ensures the patient the most correct diagnostic and therapeutic work-up.

Our choice to use the more accurate invasive biopsy method (DBT-VAB) instead of MRI is connected to the low capability of the second one to exclude the presence of associated malignancy in B3 lesions [20] with a high cost-effectiveness ratio.

Indeed, in our series, patients with architectural distortions and a B5 microhistological result have been treated with conservative surgery which was permitted by the early detection of breast cancer (with small lesion sizes) with lower costs for cancer treatment [17].

The small sample size, due to the fact that AD is not a very frequent finding and the retrospective nature of this study, has limitations.

With the growing diffusion of DBT, even outside dedicated breast imaging centres, a broad diffusion and education about DBT findings is beneficial to guarantee a great level of care to patients. Likewise, the use of DBT in breast cancer screening is strictly connected to the availability of DBT-VAB in the same hospital or in a referral centre. Thanks to the reduced overall procedure time, the low rate of complications and the low radiation exposure, DBT-VAB can also be performed in patients with abnormalities demonstrated on 2D digital mammograms, as well [21,22].

## 5. Conclusions

In conclusion, DBT only ADs demonstrated a lower PPV of malignancy when compared with synt2D mammography, and DBT detected ADs but was not low enough to avoid biopsy. A US correlate was often associated with maligna cy, and therefore should increase the level of suspicion, even when CNB yielded a B3 result.

## Figures and Tables

**Table 1:** Histopathological correlation of only DBT and DBT + synt2D detected Ads.

	B2	B3	B4	B5	TOT.
ADs only DBT detected	3 (11.5%)	18 (69.2%)	0 (0%)	5 (19.2%)	26 (23.4%)
ADs DBT and synt2D detected	16 (18.8%)	47 (55.2%)	0 (0%)	22 (25.8%)	85 (76.5%)
TOT.	19 (17.1%)	65 (58.5%)	0 (0%)	27 (24.3%)	111 (100%)

**Table 2:** Histopathological correlation of DBT + US detected ADs and for DBT + synt2D + US detected ADs.

	B2	B3	B4	B5	TOT.
ADs DBT and US detected	0 (0%)	1 (33.3%)	0 (0%)	2 (66.6%)	3 (25%)
ADs DBT, synt2D and US detected	3 (33.3%)	2 (22.2%)	0 (0%)	4 (44.4%)	9 (75%)
TOT.	3 (25%)	3 (25%)	0 (0%)	6 (50%)	12 (100%)

**Table 3:** Escissional surgical-biopsy and 2 year follow up B3 to B5 upgrading of lesions.

	Total Number of B3 Lesions for Each Group	B3 to B5 Upgrading after Surgical Biopsy	B3 to B5 Upgrading at 2 Years’ Follow Up
ADs only DBT detected	18/26 (69.2%)	0/18 (0%)	0/18 (0%)
ADs DBT and synt2D detected	47/85 (55.2%)	2/47 (4.2%)	0/47 (0%)
ADs DBT and US detected	1/3 (33.3%)	1/1 (100%)	-
ADs DBT, synt2D and US detected	2/9 (22.2%)	½ (50%)	0/2 (0%)

## Data Availability

The data presented in this study are available on request from the corresponding author. The data are not publicly available due to privacy.
